# Electrochromic Behavior of a Di-μ-Phenoxo-Bridged Iron(III) Salen-Based Complex: A Combined Electrochemical and Spectroelectrochemical Study

**DOI:** 10.3390/ijms27156714

**Published:** 2026-07-27

**Authors:** Sergiusz Napierała, Mateusz Bogusławski, Maciej Kubicki, Monika Wałęsa-Chorab

**Affiliations:** Faculty of Chemistry, Adam Mickiewicz University in Poznan, Uniwersytetu Poznanskiego 8, 61-614 Poznan, Poland

**Keywords:** di-μ-phenoxo-bridged iron(III) complex, salen ligand, Schiff base, tetraphenylethylene, electrochromism, spectroelectrochemistry, cyclic voltammetry

## Abstract

A tetraphenylethylene-based salen-type Schiff base ligand and its Fe(III) coordination complex were synthesized and characterized using spectroscopic, electrochemical, mass spectrometric, and single-crystal X-ray diffraction techniques. The ligand features an N_2_O_2_ donor set and was obtained via Schiff base condensation with ethylenediamine, while reaction with FeCl_3_ afforded a di-μ-phenoxo-bridged dinuclear Fe(III) complex under mild conditions. Unlike previously reported Fe(III)-salen electrochromic systems, which are predominantly mononuclear and exhibit ligand-centered redox processes with limited modulation of intraligand electronic communication, the present system incorporates a rigid tetraphenylethylene scaffold and forms a centrosymmetric di-μ-phenoxo-bridged Fe(III) dimer. This structural motif enables coordination-induced electronic coupling between phenolate units, resulting in a distinct splitting of ligand-centered oxidation processes. Single-crystal X-ray diffraction confirmed a di-μ-phenoxo-bridged Fe(III) dimer with distorted octahedral geometry. Electrochemical studies show a quasi-reversible ligand-centered oxidation in the free ligand, which splits into two separate redox events upon complexation, indicating the emergence of electronically non-equivalent redox sites. Spectroelectrochemical analysis reveals the formation of phenoxyl radical species accompanied by ligand-centered intervalence charge–transfer transitions and the appearance of a near-infrared absorption band upon oxidation. A reversible color change from red to blue is observed, reflecting redox-driven modulation of the electronic structure. Overall, this work demonstrates that incorporation of a tetraphenylethylene-based salen framework and formation of a di-μ-phenoxo-bridged Fe(III) dimer enables coordination-triggered intraligand electronic communication, leading to fundamentally different redox behavior compared to previously reported Fe(III)-salen electrochromic complexes, while no reversible metal-centered redox processes were detected within the experimentally investigated potential window.

## 1. Introduction

Salen-type ligands are widely recognized in coordination chemistry for their structural rigidity and reliability as chelating agents [1,2]. Their synthesis is quite straightforward and modular, which makes them appealing candidates to use as ligands during complexation with transition-metal ions [3,4]. Their N_2_O_2_ donor moiety provides a well-defined coordination environment that can stabilize metals with different oxidation states. Because of this stability, the salen-type complexes have been studied extensively in areas such as catalysis [5,6], redox reactions [7], and molecular electronics [8,9,10]. Among them, iron(III) salen complexes have drawn particular attention, often serving as model compounds to investigate electron-transfer mechanisms [11], magnetic properties [11], and relationships between structure and function [6,12,13].

Adjusting the physicochemical characteristics of these metal complexes often involves introducing extended organic frameworks into the salen ligands. Tetraphenylethylene-based units are especially interesting in this context [14]. Their bulky, non-planar shape influences not just how metal centers interact electronically, but also the redox properties of the entire complex [15]. The three-dimensional structure of tetraphenylethylene derivatives tends to prevent tight packing and π-π stacking interactions, but at the same time, they offer a conjugated backbone that can shift electron density during oxidation or reduction [15]. This combination makes tetraphenylethylene-containing ligands quite useful for synthesis and tuning redox-active coordination compounds [16,17].

Complexes of iron(III) with salen-type Schiff base ligands continue to attract considerable attention due to their structural versatility of the salen platform. These compounds also exhibit rich redox chemistry involving both metal-centered Fe(III)/Fe(II) couples and ligand-centered oxidation processes [11,18]. In particular, the N_2_O_2_ salen scaffold provides a strong chelating environment while axial ligands and/or bridging oxygen donors commonly complete five- or six-coordinate, square-pyramidal or distorted-octahedral geometries around Fe(III) [11,19]. A recurring structural motif in Fe(III)-salen chemistry is the formation of di-μ-phenoxo-bridged dinuclear species [20], in which the phenoxo bridges mediate magnetic superexchange and influence the electrochemical response of the metal–ligand framework [11,19,21]. Such bridging motifs may facilitate electronic communication between the two coordination units, while ligand substitution and axial coordination modulate the potentials and localization of metal- and ligand-centered redox processes [11,18]. As such, di-μ-phenoxo-bridged dinuclear iron-salen compounds are valuable for studying how changes in ligand design and metal nuclearity impact electrochemical behavior and charge transfer.

Electrochemical tools, often used alongside spectroelectrochemical methods, reveal detailed information about redox processes and the related electronic and structural transformations in these complexes. Gaining a solid grasp of these changes is key for designing coordination compounds that show reversible redox-driven optical shifts, like those found in electrochromic materials. In this light, iron(III) salen complexes that incorporate tetraphenylethylene-based ligands stand out as promising examples, offering insights into the interplay between molecular structure and electrochemical performance in non-luminescent, redox-active systems.

Despite the large number of reported Fe(III)-salen complexes, including di-μ-phenoxo-bridged dinuclear architectures, electrochemical and electrochromic studies have largely focused on systems based on relatively simple salicylidene frameworks [11,18,19,21,22]. Representative studies of phenoxo-bridged Fe(III) dimers have mainly addressed their synthesis, molecular structure, magnetic coupling, and metal-centered redox behavior rather than the influence of extended π-conjugated scaffolds on intraligand electronic communication [11,19,21]. Consequently, the effect of extended π-conjugation on communication between phenolate units in di-μ-phenoxo-bridged Fe(III)-salen dimers remains insufficiently explored. Systematic spectroelectrochemical studies correlating structural constraints with the appearance of intervalence charge–transfer features in oxidized states are likewise scarce [22]. As a result, the role of ligand architecture in governing stepwise oxidation processes and electronic asymmetry in dinuclear Fe(III)-salen systems remains an open question.

**Figure 1:**
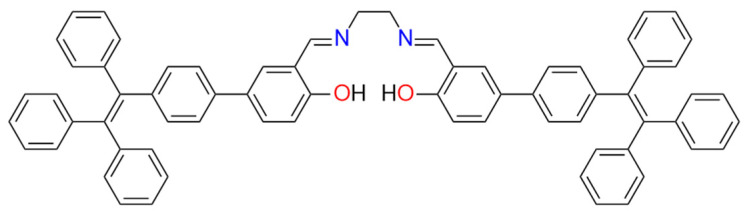
Schematic representation of ligand **L**.

Herein, we present a salen-type ligand bearing two symmetrically substituted tetraphenylethylene units (Figure 1) and its corresponding dinuclear di-μ-phenoxo-bridged Fe(III) complex, obtained via a straightforward direct coordination approach. In our previous study, we reported the template-assisted synthesis of analogous Zn(II), Ni(II), and Cu(II) complexes based on the same ligand framework, which afforded exclusively mononuclear species adopting square-planar geometries [14]. In contrast, the present work highlights the distinct coordination behavior of Fe(III), whose intrinsic coordination preferences disfavor square-planar environments and instead promote octahedral coordination, ultimately leading to the formation of a di-μ-phenoxo-bridged- dinuclear architecture.

Owing to the redox activity of the salen-based ligand scaffold, the resulting complex was further investigated with respect to its electrochemical and spectroelectrochemical properties.

## 2. Results and Discussion

### 2.1. Synthesis and Characterization of the Complex

**Figure 2:**
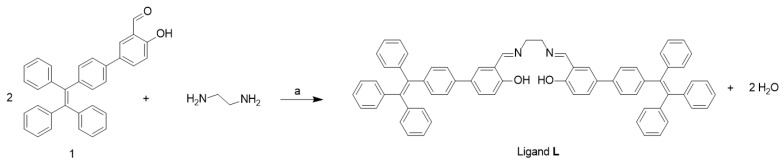
A synthesis scheme of ligand **L**; conditions: **a.** absolute ethanol, 70 °C, 48 h.

The synthesis of the target tetraphenylethylene-based ligand was designed to provide a rigid π-conjugated scaffold bearing multiple coordination sites suitable for subsequent coordination chemistry studies. To achieve this, a concise multistep synthetic strategy involving sequential halogenation, borylation, and palladium-catalyzed cross-coupling reactions [23,24,25,26,27,28,29] was employed to obtain aldehyde derivative **1**, as described previously [14]. Ligand **L** was subsequently obtained via a Schiff base condensation reaction between compound **1** and ethylenediamine under inert conditions in ethanol (Figure 2).

The reaction mixture was heated under reflux to promote efficient imine formation and ensure complete conversion of the aldehyde functionalities. The formation of the product led to a more rigid molecular architecture incorporating flexible aliphatic linkers derived from ethylenediamine, rather than an extended π-conjugated system. After completion of the reaction, the solution was concentrated under reduced pressure, and the crude product was precipitated by cooling in an ice bath to facilitate isolation of the organic product. The resulting yellow solid was collected, thoroughly washed with ethanol to remove residual starting materials and by-products, and dried under vacuum to afford ligand **L** in good yield. The obtained ligand features an N_2_O_2_ donor set composed of two imine nitrogen atoms and two phenolic oxygen atoms, providing an effective binding motif for metal ion coordination in subsequent complexation studies.

The Fe(III) complex was synthesized via a template coordination approach using ligand **L** and iron(III) chloride in a mixed dichloromethane/acetonitrile medium under ambient conditions. After addition of the Fe(III) precursor to the ligand solution, an immediate color change to an intense red was observed, which suggested rapid coordination of the metal center and formation of the corresponding complex species in solution. The reaction mixture was subsequently stirred at room temperature for 24 h to ensure complete metal incorporation and stabilization of the coordination environment around the Fe(III) ion. Following completion of the reaction, the solution was concentrated under reduced pressure, and the product was precipitated with diethyl ether, affording the Fe(III) complex as a red solid. The obtained complex exhibited good stability under ambient conditions and remained readily soluble in common polar organic solvents, which facilitated its further characterization. The immediate appearance of an intense red-purple coloration upon addition of Fe(III) is consistent with coordination-driven charge–transfer processes commonly observed in Fe(III) Schiff base complexes [30,31] and additionally supports successful complex formation. The adopted synthetic strategy proved to be straightforward and efficient, providing the desired coordination compound in analytically pure form suitable for subsequent spectroscopic and structural investigations.

The ligand **L** and its corresponding Fe(III) complex were comprehensively characterized using high-resolution electrospray ionization mass spectrometry (HR-ESI-MS) and Fourier-transform infrared spectroscopy (FT-IR) (Figures S2–S6). The combination of these analytical techniques provided detailed insight into the molecular composition of the obtained compounds, the coordination mode of the ligand, and the oxidation state of the metal centers. The HR-ESI-MS spectrum of ligand **L** displayed a prominent molecular ion peak at *m*/*z* 929.4102 (Figure S5), which was in excellent agreement with the calculated value for the proposed molecular formula. In the case of the Fe(III) complex, the mass spectrum revealed the formation of a species with the formula [Fe**L**]^+^ at *m*/*z* 982.3227 (Figure S6). However, the isotopic pattern of this signal corresponds to a mononuclear [Fe**L**]^+^ species rather than a dimeric [Fe_2_**L**_2_]^2+^ assembly, suggesting that the dimer undergoes fragmentation under ESI-MS conditions. This behavior is commonly observed for coordination compounds of this type, where non-covalent or labile metal–ligand interactions can lead to dissociation in the gas phase during electrospray ionization [32].

Further structural information was obtained from FT-IR spectroscopy, which proved highly useful for evaluating the coordination behavior of the ligand and identifying characteristic metal–ligand interactions (Figure S4). In the FT-IR spectrum of ligand **L**, no distinct O-H stretching band in the region around 3000–3500 cm^−1^ was observed. The absence of a well-defined phenolic O-H band can be attributed to strong intramolecular O-H⋯N hydrogen bonding, which is characteristic of Schiff base ligands derived from salicylaldehydes. This interaction leads to the formation of a chelated hydrogen-bonded O-H group, resulting in a very broad and often weak absorption in the 2500–3000 cm^−1^ region, which may be significantly broadened or masked in the IR spectrum [33]. However, the presence of phenolic protons in the ligand structure was confirmed by ^1^H NMR spectroscopy, which showed a characteristic downfield-shifted signal at δ = 13.44 ppm (Figure S2), consistent with strong intramolecular O-H⋯N hydrogen bonding typically observed in salicylidene-derived Schiff base (salen-type) ligands [34]. Comparison of the FT-IR spectra of the free ligand and the Fe(III) complex revealed several significant changes associated with the coordination process. The ν(C=N) stretching vibration of the imine group in the free ligand was observed at 1629 cm^−1^. Upon coordination to the Fe(III) centers, this band shifted to lower wavenumbers and split into two distinct signals at 1620 and 1602 cm^−1^. This splitting is attributed to the formation of nonequivalent imine environments resulting from asymmetric coordination of the ligand to two Fe(III) centers, which is consistent with the crystallographically determined binuclear structure. The presence of a band at 820 cm^−1^ is attributed to Fe-O-Fe stretching vibrations [35].

**Figure 3:**
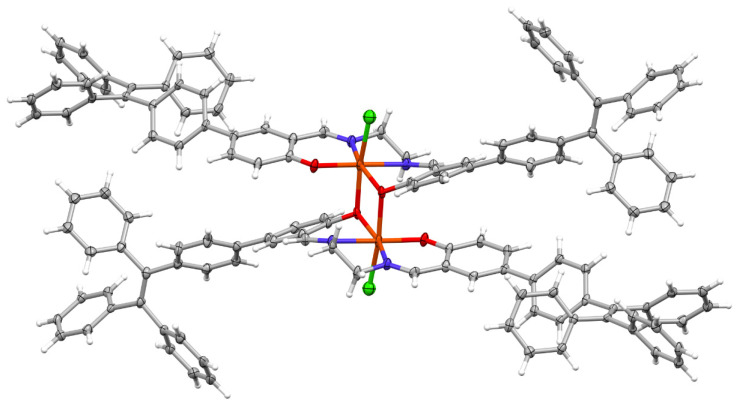
Perspective view of the Fe(III) complex. Ellipsoids are drawn at the 50% probability level; hydrogen atoms are represented by spheres of arbitrary radii.

While HR-ESI-MS and FT-IR analyses provide strong evidence for the formation of the target Fe(III) coordination compound and its proposed binding mode, unambiguous verification of its nuclearity and precise geometric structure was achieved through single-crystal X-ray diffraction analysis. The single crystal suitable for X-ray analysis was grown by slow diffusion of diethyl ether into the solution of the complex in acetonitrile. Figure 3 shows the perspective view of the complex molecule. The complex in the solid state is *C_i_*-symmetrical (lying across the inversion center in the space group P-1), two-centered, and di-O-bridged. The Fe centers are six-coordinated in the distorted octahedral geometry (cf. Table 1 for the relevant geometrical parameters). In the crystal structure there are voids (Figure S1) which account for more than 21% of the unit cell volume, which are filled by the solvent guest–acetonitrile molecules.

### 2.2. Electrochemical Investigation

**Figure 4:**
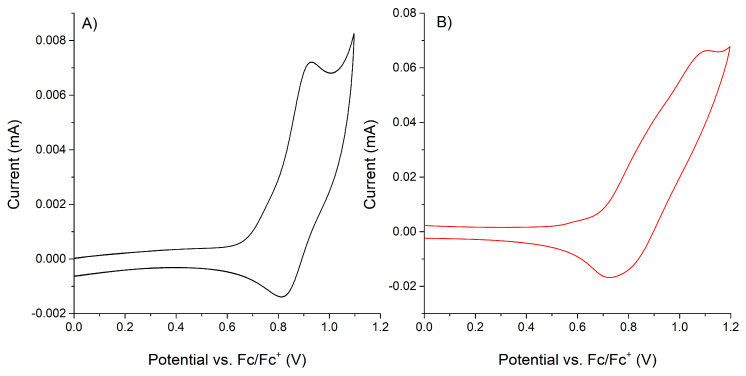
Cyclic voltammograms of the ligand **L** (**A**) and Fe(III) complex (**B**) measured in an anhydrous and deaerated 0.1 M solution of TBAPF_6_ in dichloromethane, using a three-electrode system, at a scan rate of 100 mV s^−1^.

To evaluate the potential usability of the compounds as electroactive materials, ligand **L** and the Fe(III) complex were investigated with respect to their electrochemical response to applied electrical stimuli. For this purpose, cyclic voltammetry experiments were performed using a three-electrode system consisting of a platinum working electrode (ϕ = 2 mm), a non-aqueous silver Ag/Ag^+^ reference electrode and a platinum wire as the counter electrode. An anhydrous and deaerated 0.1 M solution of tetrabutylammonium hexafluorophosphate in dry dichloromethane was used as the supporting electrolyte. All measured potentials were calibrated against the ferrocene/ferrocenium (Fc/Fc^+^) redox couple [36], providing a consistent reference point for accurate comparison of the observed redox processes. The cyclic voltammograms of ligand **L** and the iron(III) complex are presented in Figure 4.

The electrochemical behavior of ligand **L** was investigated by cyclic voltammetry in dichloromethane containing 0.1 M TBAPF_6_ as the supporting electrolyte. The voltammogram of free ligand **L** (Figure 4A) exhibited an anodic wave at E_pa_ = +0.93 V, accompanied by a corresponding cathodic signal at E_pc_ = +0.81 V, indicating a quasi-reversible redox process. This redox event can be attributed to a ligand-centered oxidation process involving the phenolate moieties of the salen-type framework, leading to the formation of a phenoxyl radical species. Notably, only a single redox couple was observed despite the presence of two phenolic donor groups within the ligand structure. According to the literature, electronically coupled phenolate units within salen-type frameworks typically undergo stepwise ligand-centered oxidation processes, resulting in two distinct redox processes. In contrast, the observation of only one oxidation wave suggests that the two phenolate centers in ligand **L** are electrochemically equivalent and exhibit negligible electronic communication. Consequently, oxidation of one phenolate unit does not significantly influence the redox behavior of the second one, indicating that the two redox-active sites behave independently. Such behavior is consistent with a limited degree of electronic delocalization across the ligand framework and the absence of efficient electronic coupling between the phenolate donor groups. This assignment was further confirmed by differential pulse voltammetry (DPV) (Figure S7A), which reproduced a single well-defined oxidation signal at the same potential, supporting the conclusion of electrochemically equivalent and non-interacting phenolate sites within the ligand.

The cyclic voltammogram recorded for the Fe(III) complex (Figure 4B) revealed a significantly different redox response compared to that of the free ligand. In the anodic region, a broad oxidation wave was observed at E_pa_ = +1.10 V, accompanied by a corresponding cathodic peak at E_pc_ = +0.73 V. The shape and width of this redox feature suggest the overlap of multiple closely spaced processes. This interpretation was confirmed by DPV measurements (Figure S7), which showed a single oxidation event for ligand **L**, while two well-separated oxidation processes were resolved for the Fe(III) complex, demonstrating the splitting of the ligand-centered oxidation upon coordination. The splitting of the oxidation processes, in contrast to the single wave observed for the free ligand, indicates that coordination to Fe(III) modifies the electronic interaction between the coordinated phenolate moieties of the salen framework, leading to non-equivalent redox behavior of the donor groups upon metal binding. The first oxidation process can be assigned to the formation of a ligand-centered radical cation, whereas the second oxidation step corresponds to the generation of the dicationic species [14,18]. Within the investigated potential window, down to −1.0 V no well-defined or reversible Fe(III)/Fe(II) redox couple was observed. For Fe(III)-salen-type complexes, this process is typically located at significantly more negative potentials [37,38,39] than those commonly observed for iron complexes bearing other classes of ligands [40,41,42,43,44,45] and may therefore fall outside the accessible electrochemical window in acetonitrile or be obscured by ligand-centered processes.

Overall, the electrochemical data clearly demonstrates a transition from an electronically isolated ligand system in the free state to a metal-coordinated framework in which the phenolate units become electronically coupled. This coupling leads to a more complex, stepwise oxidation pattern and highlights the strong influence of Fe(III) coordination on the redox properties of the salen scaffold, while iron-centered redox chemistry was not observed within the investigated potential window.

In classical transition-metal salen complexes, electrochemical oxidation of the phenolate moieties frequently generates ligand-centered phenoxyl radicals, which may subsequently undergo intermolecular aryl-aryl (C-C) coupling, resulting in the formation of electroactive poly(salen) films on the electrode surface [46,47,48]. Such electropolymerization generally proceeds through the unsubstituted 5-position (para to the phenolate oxygen) of the salicylidene ring [49,50]. Consequently, the availability of these reactive positions plays a decisive role in determining whether oxidative polymerization can occur [51]. In the present ligand framework, the salicylidene rings are substituted with bulky tetraphenylethylene groups, which block the principal coupling sites and sterically hinder intermolecular C-C bond formation. This design strategy was previously shown to suppress electropolymerization in the analogous Cu(II), Ni(II), and Zn(II) complexes based on the same ligand scaffold, for which repeated cyclic voltammetry experiments revealed no evidence of polymer film growth [14]. Although analogous electropolymerization studies were not performed for the Fe(III) complex described herein, its identical ligand architecture suggests that the sterically blocked coupling positions should likewise disfavor oxidative polymer growth.

### 2.3. Spectroelectrochemical Properties

**Figure 5:**
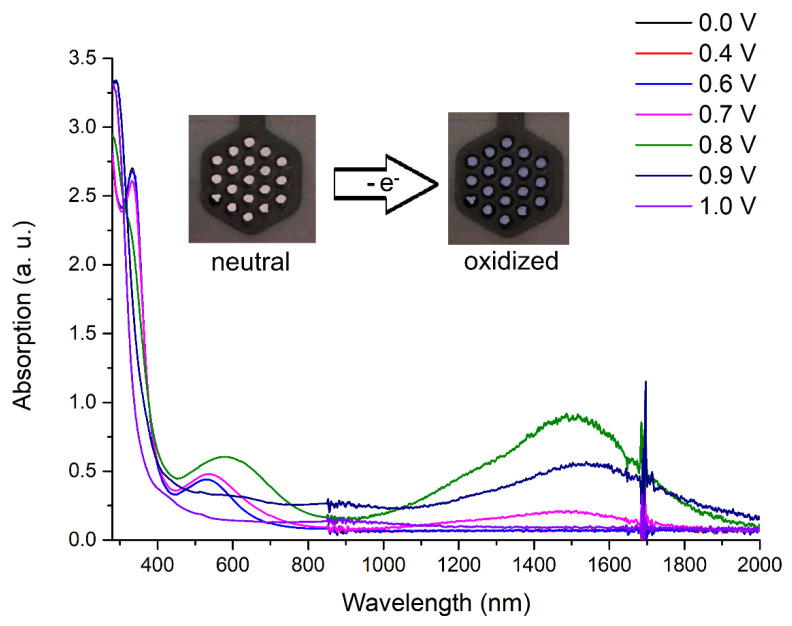
Spectroelectrochemistry of the Fe(III) complex recorded in an anhydrous and deaerated 0.1 M solution of TBAPF_6_ in dichloromethane as the supporting electrolyte, referenced to the Fc/Fc^+^ redox couple, together with photographs illustrating the corresponding color changes of the different redox states.

To investigate the electrochromic behavior of the synthesized ligand and complex, spectroelectrochemical tests were performed, as shown in Figure S8 and Figure 5. The data demonstrates that the color and electrochemical response of the compounds change significantly upon complexation and the addition of iron(III) ions. They display distinctive color changes when an external electric potential is applied. All tests were performed in a solution of 0.1 M tetrabutylammonium hexafluorophosphate (TBAPF_6_) dissolved in dichloromethane, which served as both the supporting electrolyte and the solvent.

For ligand **L** (Figure S8), the UV-Vis spectrum in the neutral state is dominated by absorption bands at 290 nm and 330 nm, which are characteristic of π-π* and intraligand charge–transfer transitions of the unoxidized system (Figure S8) [18,52]. Upon gradual increase of the applied anodic potential, only minor changes in the UV-Vis spectra were observed. These changes were limited to slight variations in the intensity of the absorption bands and did not result in any noticeable shift of the absorption maxima or affect the observed color of the solution. The spectroelectrochemical data therefore indicate that oxidation of the ligand proceeds with only subtle modifications of its electronic structure under the applied experimental conditions.

For the Fe(III) complex (Figure 5), the spectroelectrochemical response differs markedly from that of the free ligand and reflects the influence of metal coordination on the electronic structure of the salen framework. In the neutral state, the absorption spectrum is characterized by two main bands at 334 nm and 530 nm. The band at 334 nm is attributed predominantly to ligand-centered π-π* and intraligand charge–transfer transitions, whereas the band at 530 nm is assigned to ligand-to-metal charge–transfer (LMCT) transitions involving electron donation from the phenolate oxygen atoms to the Fe(III) center and/or ligand-field related transitions typical for Fe(III)-salen-type coordination environments [53,54]. Upon progressive anodic polarization, significant spectral changes are observed. The intensity of the band at 334 nm gradually decreases, accompanied by the emergence of a new absorption feature at approximately 290 nm, indicating a redistribution of the ligand-centered electronic structure upon oxidation. Concurrently, a broad absorption band appears in the near-infrared (NIR) region at ca. 1500 nm, which suggests the formation of ligand-centered radical species generated during the one-electron oxidation process. These spectral changes are consistent with intervalence-like ligand-centered charge–transfer transitions, suggesting enhanced electronic delocalization within the oxidized ligand framework. The appearance of new absorption features in the 400–500 nm region and in the NIR range is consistent with the assignment of the spectroelectrochemical processes observed at the first anodic wave in the voltammetric response. In addition, the band initially observed at 530 nm undergoes a noticeable bathochromic shift and broadening, moving to approximately 585 nm. This red shift suggests a change in the electronic environment of the Fe(III) center and increased delocalization within the metal–ligand framework upon oxidation, consistent with the formation of an oxidized, more electronically coupled system. The overall spectral evolution is accompanied by a pronounced and visually observable color change of the solution from red in the neutral state to blue upon oxidation, consistent with the formation of the oxidized, radical-containing species.

Comparison with previously reported dinuclear iron Schiff base and related metal-phenoxide systems shows that their redox chemistry can involve metal-centered changes in the iron oxidation states as well as ligand-centered oxidation of coordinated phenoxide units [55,56,57]. In bis(phenoxo)-bridged diiron complexes, the Fe_2_O_2_ core provides a pathway for magnetic exchange and electronic communication between the two iron sites [56,58]. Such communication becomes particularly evident upon formation of mixed-valence Fe(III)Fe(II) species, which may exhibit either valence-trapped or valence-delocalized electronic structures depending on the ligand architecture and donor environment [55,57,59]. In the present complex, the near-infrared absorption generated upon oxidation is therefore consistent with the formation of an electronically coupled oxidized ligand state. Although the combined electrochemical and spectroelectrochemical data support this interpretation, complementary EPR spectroscopy and/or computational studies would provide additional confirmation of the electronic structure of the oxidized species. However, despite these well-established ligand-centered processes and occasional metal-involved redox chemistry, the formation of strongly delocalized intervalence charge–transfer states extending into the near-infrared region remains uncommon in classical Fe(III)-salen systems [22].

### 2.4. Accession Codes

CCDC 2524692 contains the supplementary crystallographic data for this paper. These data can be obtained free of charge via www.ccdc.cam.ac.uk/data_request/cif (accessed on 20 July 2026), or by emailing data_request@ccdc.cam.ac.uk, or by contacting The Cambridge Crystallographic Data Centre, 12 Union Road, Cambridge CB2 1EZ, UK; fax: +44 1223 336033.

## 3. Materials and Methods

### 3.1. Crystal Data

C_136_H_100_Cl_2_Fe_2_N_4_O_4_ · 6(C_2_H_3_N), M_r_ = 2283.12, triclinic, P-1, a = 9.1526(4) Å, b = 14.1551(7) Å, c = 24.4291(11) Å, α = 98.687(3)°, β = 96.638(3)°, γ = 104.288(3)°, V = 2993.0(2) Å^3^, Z = 1, d_x_ = 1.267 g·cm^−3^, F(000) = 1194, μ = 2.834 cm^−1^, 35,635 reflection collected, 10,765 symmetry independent (R_int_ = 3.79%), 9146 with I > 2σ(I). Final R [I > 2σ(I)] = 0.0710, wR2 [I > 2σ(I)] = 0.1483, R [all reflections] = 0.0907, wR2 [all reflections] = 0.1636, S = 1.089, (Δρ_max_/Δρ_min_) = 1.53/−0.81 e·Å^−3^. CCDC deposition number: 2524692.

### 3.2. Synthetic Procedures

Salicylaldehyde derivative **1** was obtained as described previously [14].

#### Ligand **L**

Compound **1** (50 mg, 0.11 mmol) was placed in a single-neck round-bottom flask. The system was evacuated and backfilled with argon to establish an inert atmosphere. Absolute ethanol (20 mL) was then added, and the mixture was heated to 30 °C to ensure complete dissolution of the substrate. Subsequently, ethylenediamine (3.69 µL) was added dropwise. The reaction mixture was stirred at 70 °C for 48 h. After completion, the solvent was removed under reduced pressure, and the resulting residue was poured onto ice. The precipitated solid was collected by centrifugation, washed several times with ethanol, and dried in a vacuum desiccator. Ligand **L** was obtained as a yellow solid (40 mg, yield: 78%). ^1^H NMR (600 MHz, DMSO) δ 13.44 (s, 2H), 8.64 (s, 2H), 7.73 (d, *J* = 2.4 Hz, 2H), 7.59 (dd, *J* = 8.6, 2.4 Hz, 2H), 7.43–7.38 (m, 4H), 7.19–7.07 (m, 18H), 7.05–7.00 (m, 12H), 6.99–6.95 (m, 4H), 6.90 (d, *J* = 8.6 Hz, 2H), 3.94 (s, 4H) ppm. ^13^C NMR (151 MHz, DMSO) δ 167.0, 160.3, 143.2, 143.2, 143.2, 141.6, 140.6, 140.1, 137.1, 131.3, 130.7, 130.6, 130.4, 129.8, 129.4, 127.9, 127.8, 127.8, 126.6, 126.6, 126.5, 125.2, 118.7, 117.1, 58.7 ppm. HR-ESI-MS: *m*/*z* calcd for C_68_H_52_N_2_O_2_ [**L**]: 929.4062 found 929.4102; ATR-FT-IR: ν(C-H)_ar_ 3078, 3052, 3023; ν(C=N)_imine_ 1629; ν(C=C)_ar_ 1592, 1558, 1545; ν(C-N), δ(C=N) 1481, 1440, 1366; ν(C-O)_phenolate_ 1297, 1278; ν(C-C)_ar_ 1177, 1128; ρ(C-H)_ar_ 856, 824, 811; γ(C-H)_ar_ 779, 768, 745, 690, 633, 617, 600 cm^−1^. Elemental analysis calcd. for C_68_H_52_N_2_O_2_ calcd. C, 87.90; H, 5.64; N, 3.01; found C, 87.92; H, 5.67; N, 3.02.

### 3.3. Fe(III) Complex

Ligand **L** (11 mg, 0.012 mmol) was introduced into a single-neck round-bottom flask and dissolved in 3 mL of a dichloromethane/acetonitrile mixture (1:1, *v*/*v*). After complete dissolution of the ligand, FeCl_3_·6H_2_O (2.8 mg, 0.010 mmol) was added. Upon addition of the iron(III) salt, the solution turned red. The reaction mixture was stirred at room temperature for 24 h. The final product was isolated by concentrating the reaction mixture under reduced pressure, followed by precipitation with diethyl ether. A red solid corresponding to the Fe(III) complex was obtained (11 mg, yield 87%). HR-ESI-MS: *m*/*z* calcd for C_68_H_50_FeN_2_O_2_^+^: 982.3217; found: 982.3227. ATR-FT-IR: ν(C-H)_ar_ 3019, 3009; ν(C=N)_imine_ 1620, 1602; ν(C=C)_ar_ 1602, 1539, 1533; ν(C-N), δ(C=N) 1491, 1465, 1443, 1378; ν(C-O)_phenolate_ 1311, 1278; ν(C-C)_ar_ 1177, 1074, 1032, 1016; ρ(C-H)_ar_ 851, 836; ν(Fe-O-Fe) 820; γ(C-H)_ar_ 774, 763, 759, 697, 633, 617, 600 cm^−1^. Elemental analysis calcd. for C_136_H_100_Cl_2_Fe_2_N_4_O_4_ calcd. C, 80.19; H, 4.95; N, 2.75; found C, 80.21; H, 4.90; N, 2.74.

## 4. Conclusions

A tetraphenylethylene-based salen-type Schiff base ligand and its di-μ-phenoxo-bridged dinuclear Fe(III) complex were successfully synthesized and structurally characterized. The combination of single-crystal X-ray diffraction, electrochemical analysis, and spectroelectrochemical measurements provides a comprehensive understanding of their redox and electronic properties.

The key novelty of this work lies in demonstrating that incorporation of a rigid tetraphenylethylene framework into a salen-type ligand, together with formation of a di-μ-phenoxo-bridged Fe(III) dimer, induces coordination-controlled intraligand electronic communication that is not observed in the corresponding free ligand. In contrast to previously reported Fe(III)-salen systems, in which ligand-centered oxidation is often observed without clearly resolved differentiation of the two phenolate redox sites, the present system displays a clear splitting of oxidation events upon metal coordination, evidencing the generation of non-equivalent redox centers and enhanced intraligand coupling. Spectroelectrochemical studies further reveal the formation of phenoxyl radical species and intervalence charge–transfer transitions in the near-infrared region, accompanied by a pronounced and reversible electrochromic response. These features highlight a direct correlation between ligand architecture, nuclearity, and redox behavior in Fe(III)-salen systems.

Overall, this study provides new insight into how extended π-conjugated salen frameworks and di-μ-phenoxo-bridged dimer formation can be used to modulate intraligand electronic interactions and redox properties. These findings expand the current understanding of structure–electronic property relationships in Fe(III)-salen coordination chemistry and may guide the rational design of next-generation redox-active and electrochromic molecular materials.

## Figures and Tables

**Table 1:** Relevant geometric parameters (Å, °) with s.u.’s in parentheses. Symmetry codes: ^i^ 1-x, 1-y, 1-z.

Fe1-N1	2.121(3)	Fe1-O7	1.899(3)
Fe1-N4	2.127(3)	Fe1-O40	2.005(2)
Fe1-O40 ^i^	2.158(3)	Fe1-Cl1	2.3345(12)
N1-Fe1-O7	160.94(12)	N4-Fe1-O40	159.42(11)
Cl1-Fe1-O40 ^i^	168.20(7)		

## Data Availability

Crystallographic data for the structural analysis have been deposited with the Cambridge Crystallographic Data Centre under deposition number CCDC 2524692. Copies of these data may be obtained free of charge from the Cambridge Crystallographic Data Centre, 12 Union Road, Cambridge CB2 1EZ, UK; e-mail: deposit@ccdc.cam.ac.uk; or at https://www.ccdc.cam.ac.uk. The original contributions presented in this study are included in the article/Supplementary Materials. Further inquiries can be directed to the corresponding author.

## Supplementary Materials

The following supporting information can be downloaded at: https://www.mdpi.com/article/10.3390/ijms27156714/s1. References [60,61,62] are cited in the Supplementary Materials.

## References

[1] Cozzi P.G. (2004). Metal–Salen Schiff Base Complexes in Catalysis: Practical Aspects. Chem. Soc. Rev..

[2] Canali L., Sherrington D.C. (1999). Utilisation of Homogeneous and Supported Chiral Metal(Salen) Complexes in Asymmetric Catalysis. Chem. Soc. Rev..

[3] Jacobsen E.N. (2000). Asymmetric Catalysis of Epoxide Ring-Opening Reactions. Acc. Chem. Res..

[4] Katsuki T. (1995). Catalytic Asymmetric Oxidations Using Optically Active (Salen)Manganese(III) Complexes as Catalysts. Coord. Chem. Rev..

[5] Duan R., Hu C., Li X., Pang X., Sun Z., Chen X., Wang X. (2017). Air-Stable Salen–Iron Complexes: Stereoselective Catalysts for Lactide and ε-Caprolactone Polymerization through in Situ Initiation. Macromolecules.

[6] Cristóvão B., Osypiuk D., Bartyzel A. (2025). Exploration of Ruthenium(II/III/VI)–Salen Complexes: From Synthesis to Functional Applications. Molecules.

[7] McCabe C.A., Mitchell A.L., Bonitatibus P.J., Dinolfo P.H. (2025). Ligand-Dependent Redox-Coupled Spin Crossover of a Five Coordinate Cobalt Salen Complex. Inorg. Chim. Acta.

[8] Kubo K., Shiga T., Yamamoto T., Tajima A., Moriwaki T., Ikemoto Y., Yamashita M., Sessini E., Mercuri M.L., Deplano P. (2011). Electronic State of a Conducting Single Molecule Magnet Based on Mn-Salen Type and Ni-Dithiolene Complexes. Inorg. Chem..

[9] Koyama K., Iijima K., Yoo D., Mori T. (2020). Transistor Properties of Salen-Type Metal Complexes. RSC Adv..

[10] Freire C., Nunes M., Pereira C., Fernandes D.M., Peixoto A.F., Rocha M. (2019). Metallo(Salen) Complexes as Versatile Building Blocks for the Fabrication of Molecular Materials and Devices with Tuned Properties. Coord. Chem. Rev..

[11] Rigamonti L., Zardi P., Carlino S., Demartin F., Castellano C., Pigani L., Ponti A., Ferretti A.M., Pasini A. (2020). Selective Formation, Reactivity, Redox and Magnetic Properties of MnIII and FeIII Dinuclear Complexes with Shortened Salen-Type Schiff Base Ligands. Int. J. Mol. Sci..

[12] Sivasubramanian V.K., Ganesan M., Rajagopal S., Ramaraj R. (2002). Iron(III)−Salen Complexes as Enzyme Models: Mechanistic Study of Oxo(Salen)Iron Complexes Oxygenation of Organic Sulfides. J. Org. Chem..

[13] Ghanbari Z., Housaindokht M.R., Izadyar M., Bozorgmehr M.R., Eshtiagh-Hosseini H., Bahrami A.R., Matin M.M., Khoshkholgh M.J. (2014). Structure-Activity Relationship for Fe(III)-Salen-Like Complexes as Potent Anticancer Agents. Sci. World J..

[14] Napierała S., Kubicki M., Wałęsa-Chorab M. (2026). Tetraphenylethylene-Substituted Salen Complexes of Cu(II), Zn(II), and Ni(II): Structural, Photophysical, Electrochromic and Electrofluorochromic Properties. J. Mol. Liq..

[15] Li D.-M., Zuo R., Wang J., Le Z. (2025). The Designs and Applications of Tetraphenylethylene Macrocycles and Cages. Chem.-Eur. J..

[16] Villa M., Ceroni P., Fermi A. (2022). Tetrachromophoric Systems Based on Rigid Tetraphenylmethane (TPM) and Tetraphenylethylene (TPE) Scaffolds. ChemPlusChem.

[17] Wang S.-C., Pan Q., Pan M. (2025). Tailored Synthetic Strategies for Tetraphenylethylene-Based Metal–Organic Frameworks: Design, Construction, and Control. Chem. Synth..

[18] Clarke R.M., Herasymchuk K., Storr T. (2017). Electronic Structure Elucidation in Oxidized Metal–Salen Complexes. Coord. Chem. Rev..

[19] Middya P., Konar A., Chattopadhyay S. (2024). An Overview of the Synthesis, Structures and Magnetic Properties of Dinuclear Iron Complexes with Salen Type Schiff Base Ligands. J. Mol. Struct..

[20] Shutilov I.D., Volodin P.A., Ovsyannikov A.S., Pyataev A.V., Frantsuzova L.V., Gerasimova D.P., Khamatgalimov A.R., Solovieva S.E., Antipin I.S. (2025). Intermolecular π-Stacking Stabilization of a New High-M/L-Ratio Coordination Motif for a Dinuclear Fe(III) Complex Supported by a “Salen-Type” Schiff Base Derivative of *o*-Xylylenediamine. Dalton Trans..

[21] Yu F., Cao Z.-H., Ge J.-Y., Sun Y.-C., Ouyang Z.-W., Zuo J.-L., Wang Z., Kurmoo M. (2017). Magnetostructural Relationship for μ_2_-Phenoxido Bridged Ferric Dimers. Dalton Trans..

[22] Miomandre F., Audebert P., Maumy M., Uhl L. (2001). Electrochemical Behaviour of Iron(III) Salen and Poly(Iron–Salen). Application to the Electrocatalytic Reduction of Hydrogen Peroxide and Oxygen. J. Electroanal. Chem..

[23] Xu B., Chi Z., Zhang X., Li H., Chen C., Liu S., Zhang Y., Xu J. (2011). A New Ligand and Its Complex with Multi-Stimuli-Responsive and Aggregation-Induced Emission Effects. Chem. Commun..

[24] Talloj S.K., Mohammed M., Lin H.-C. (2020). Construction of Self-Assembled Nanostructure-Based Tetraphenylethylene Dipeptides: Supramolecular Nanobelts as Biomimetic Hydrogels for Cell Adhesion and Proliferation. J. Mater. Chem. B.

[25] Chan C.Y.K., Lam J.W.Y., Zhao Z., Deng C., Chen S., Lu P., Sung H.H.Y., Kwok H.S., Ma Y., Williams I.D. (2012). A Facile Approach to Highly Efficient and Thermally Stable Solid-State Emitters: Knitting up AIE-Active TPE Luminogens by Aryl Linkers. ChemPlusChem.

[26] Hu R., Maldonado J.L., Rodriguez M., Deng C., Jim C.K.W., Lam J.W.Y., Yuen M.M.F., Ramos-Ortiz G., Tang B.Z. (2012). Luminogenic Materials Constructed from Tetraphenylethene Building Blocks: Synthesis, Aggregation-Induced Emission, Two-Photon Absorption, Light Refraction, and Explosive Detection. J. Mater. Chem..

[27] Biesen L., May L., Nirmalananthan-Budau N., Hoffmann K., Resch-Genger U., Müller T.J.J. (2021). Communication of Bichromophore Emission upon Aggregation—Aroyl-S,N-Ketene Acetals as Multifunctional Sensor Merocyanines. Chem.-Eur. J..

[28] Yu Q., Su X., Zhang T., Zhang Y.-M., Li M., Liu Y., Zhang S.X.-A. (2018). Non-Invasive Fluorescence Switch in Polymer Films Based on Spiropyran-Photoacid Modified TPE. J. Mater. Chem. C Mater..

[29] Jiang T., Fan Y., Lu J.-H., Huang C., Zhu B.-X. (2024). Two AIE-Active Schiff Base Fluorescent Probes for Highly Selective Recognition of Cu^2+^ Ions. Spectrochim. Acta A Mol. Biomol. Spectrosc..

[30] Soufeena P.P., Aravindakshan K.K. (2019). Antipyrine Derived Schiff Base: A Colurimetric Sensor for Fe(III) and “Turn-on” Fluorescent Sensor for Al(III). J. Lumin..

[31] Choi Y.W., Park G.J., Na Y.J., Jo H.Y., Lee S.A., You G.R., Kim C. (2014). A Single Schiff Base Molecule for Recognizing Multiple Metal Ions: A Fluorescence Sensor for Zn(II) and Al(III) and Colorimetric Sensor for Fe(II) and Fe(III). Sens. Actuators B Chem..

[32] Gozet T., Huynh L., Bohme D.K. (2010). Generation and Dissociation of Oxygen- and Chloride-bridged Iron(III) and Manganese(III) Tetraphenylporphyrin Dimer Ions in the Gas Phase. J. Mass Spectrom..

[33] Danilova J.S., Avdoshenko S.M., Karushev M.P., Timonov A.M., Dmitrieva E. (2021). Infrared Spectroscopic Study of Nickel Complexes with Salen-Type Ligands and Their Polymers. J. Mol. Struct..

[34] Makal A., Schilf W., Kamieński B., Szady-Chelmieniecka A., Grech E., Woźniak K. (2011). Hydrogen Bonding in Schiff Bases—NMR, Structural and Experimental Charge Density Studies. Dalton Trans..

[35] Bocian A., Napierała S., Gorczyński A., Kubicki M., Wałȩsa-Chorab M., Patroniak V. (2019). The First Example of an Asymmetrical μ-Oxo Bridged Dinuclear Iron Complex with a Terpyridine Ligand. N. J. Chem..

[36] Connelly N.G., Geiger W.E. (1996). Chemical Redox Agents for Organometallic Chemistry. Chem. Rev..

[37] Costes J.-P., Tommasino J.-B., Carré B., Soulet F., Fabre P.-L. (1995). Electrochemical Studies of Iron(III) Schiff Base Complexes—II. Dimeric μ-OXO[FeIII(N_2_O_2_)]2O Complexes. Polyhedron.

[38] Descher H., Strich S.L., Hermann M., Enoh P., Kircher B., Gust R. (2023). Investigations on the Influence of the Axial Ligand in [Salophene]Iron(III) Complexes on Biological Activity and Redox Behavior. Int. J. Mol. Sci..

[39] Chantarojsiri T., Ziller J.W., Yang J.Y. (2018). Incorporation of Redox-Inactive Cations Promotes Iron Catalyzed Aerobic C–H Oxidation at Mild Potentials. Chem. Sci..

[40] Ferreira H., von Eschwege K.G., Conradie J. (2016). Electronic Properties of Fe Charge Transfer Complexes—A Combined Experimental and Theoretical Approach. Electrochim. Acta.

[41] Banasz R., Kubicki M., Wałȩsa-Chorab M. (2020). Yellow-to-Brown and Yellow-to-Green Electrochromic Devices Based on Complexes of Transition Metal Ions with a Triphenylamine-Based Ligand. Dalton Trans..

[42] Napierała S., Kubicki M., Wałęsa-Chorab M. (2021). Toward Electrochromic Metallopolymers: Synthesis and Properties of Polyazomethines Based on Complexes of Transition-Metal Ions. Inorg. Chem..

[43] Wałȩsa-Chorab M., Banasz R., Marcinkowski D., Kubicki M., Patroniak V. (2017). Electrochromism and Electrochemical Properties of Complexes of Transition Metal Ions with Benzimidazole-Based Ligand. RSC Adv..

[44] Banasz R., Wałęsa-Chorab M. (2019). Polymeric Complexes of Transition Metal Ions as Electrochromic Materials: Synthesis and Properties. Coord. Chem. Rev..

[45] Napierała S., Muras K., Dutkiewicz G., Wałęsa-Chorab M. (2021). Reductive Electropolymerization and Electrochromism of Iron(II) Complex with Styrene-Based Ligand. Materials.

[46] Łępicka K., Pieta P., Francius G., Walcarius A., Kutner W. (2019). Structure-Reactivity Requirements with Respect to Nickel-Salen Based Polymers for Enhanced Electrochemical Stability. Electrochim. Acta.

[47] Polozhentseva J., Novozhilova M., Karushev M. (2022). Reversible Redox Processes in Polymer of Unmetalated Salen-Type Ligand: Combined Electrochemical in Situ Studies and Direct Comparison with Corresponding Nickel Metallopolymer. Int. J. Mol. Sci..

[48] Novoselova J., Lukyanov D.A., Nenova A.V., Koziakova A.V., Glumov O.V., Alekseeva E.V., Levin O.V. (2025). A Breakthrough in the Processability of NiSalen Polymers: Chemical Polymerization of NiSalens. ACS Appl. Energy Mater..

[49] Tomczyk D., Bukowski W., Bester K., Urbaniak P., Seliger P., Andrijewski G., Skrzypek S. (2017). The Mechanism of Electropolymerization of Nickel(II) Salen Type Complexes. N. J. Chem..

[50] Korusenko P.M., Petrova O.V., Vereshchagin A.A., Levin O.V., Khramov E.V., Chumakov R.G., Soldatov M.A., Katin K.P., Konev A.S., Vinogradov A.S. (2025). The Structure of the NiO_2_N_2_ Coordination Center in the [Ni(Salen)] Complex and Its Polymer: A Comparative Study by X-Ray Absorption Spectroscopy and Quantum-Chemical Calculations. Phys. Chem. Chem. Phys..

[51] Tomczyk D., Bukowski W., Bester K. (2018). Redox Processes in the Solution of Ni(II) Complex with Salen Type Ligand and in the Polymer Films. Electrochim. Acta.

[52] Kaczmarek M.T., Skrobanska M., Zabiszak M., Wałęsa-Chorab M., Kubicki M., Jastrzab R. (2018). Coordination Properties of N,N′-Bis(5-Methylsalicylidene)-2-Hydroxy-1,3-Propanediamine with d- and f-Electron Ions: Crystal Structure, Stability in Solution, Spectroscopic and Spectroelectrochemical Studies. RSC Adv..

[53] Lanznaster M., Neves A., Bortoluzzi A.J., Szpoganicz B., Schwingel E. (2002). New Fe^III^ Zn^II^ Complex Containing a Single Terminal Fe−O_phenolate_ Bond as a Structural and Functional Model for the Active Site of Red Kidney Bean Purple Acid Phosphatase. Inorg. Chem..

[54] Usman M., Hussein M.A.T., Kandiel T.A., Yamani Z.H., Shaikh M.N. (2026). Catalytic Ammonia Oxidation Mediated by High-Spin Fe(III) Complex: Combined Experimental and DFT Study. Catal. Sci. Technol..

[55] Surerus K.K., Munck E., Snyder B.S., Holm R.H. (1989). A Binuclear Mixed-Valence Ferromagnetic Iron System with an S = 9/2 Ground State and Valence Trapped and Detrapped States. J. Am. Chem. Soc..

[56] Snyder B.S., Patterson G.S., Abrahamson A.J., Holm R.H. (1989). Binuclear Iron System Ferromagnetic in Three Oxidation States: Synthesis, Structures, and Electronic Aspects of Molecules with a Fe2(OR)2 Bridge Unit Containing Fe(III,III), Fe(III,II), and Fe(II,II). J. Am. Chem. Soc..

[57] Dutta S.K., Ensling J., Werner R., Flörke U., Haase W., Gütlich P., Nag K. (1997). Valence-Delocalized and Valence-Trapped Fe^II^Fe^III^ Complexes: Drastic Influence of the Ligands. Angew. Chem. Int. Ed. Engl..

[58] Hendrich M.P., Day E.P., Wang C.-P., Synder B.S., Holm R.H., Munck E. (1994). Ground-State Electronic Structures of Binuclear Iron(II) Sites: Experimental Protocol and a Consistent Description of Moessbauer, EPR, and Magnetization Measurements of the Bis(Phenolate)-Bridged Complex [Fe2(Salmp)2]2-. Inorg. Chem..

[59] Hazra S., Sasmal S., Fleck M., Grandjean F., Sougrati M.T., Ghosh M., Harris T.D., Bonville P., Long G.J., Mohanta S. (2011). Slow Magnetic Relaxation and Electron Delocalization in an *S* = 9/2 Iron(II/III) Complex with Two Crystallographically Inequivalent Iron Sites. J. Chem. Phys..

[60] Rigaku Oxford Diffraction (2021). CrysAlis PRO.

[61] Sheldrick G.M. (2015). SHELXT–Integrated space-group and crystal-structure determination. Acta Crystallogr. A Found. Adv..

[62] Sheldrick G.M. (2015). Crystal structure refinement with SHELXL. Acta Crystallogr. C Struct. Chem..

