# Supplementary material for: Electrochromic Behavior of a Di-μ-Phenoxo-Bridged Iron(III) Salen-Based Complex: A Combined Electrochemical and Spectroelectrochemical Study
Source: Int J Mol Sci. 2026 Jul 27;27(15):6714. doi: 10.3390/ijms27156714 (PMC13466991; doi:10.3390/ijms27156714)
Supplement: Supplementary file 1 [file ijms-27-06714-s001.zip › ijms-4431656-supplementary.pdf]

Supplementary information

Electrochromic Behavior of a Di-μ-Phenoxo-Bridged Iron(III) Salen-Based Complex: A Combined Electrochemical and Spectroelectrochemical Study

Sergiusz Napierała, Mateusz Bogusławski, Maciej Kubicki, Monika Wałęsa-Chorab*

Faculty of Chemistry, Adam Mickiewicz University in Poznań, Uniwersytetu Poznańskiego 8, 61-614 Poznań, Poland

* *Correspondence: mchorab@amu.edu.pl*

Table of content

Experimental section 3

X-ray crystallography 4

Figure S1. Voids in the structure 1 (after removing toluene molecule) as seen along b-direction. 5

Figure S2. ^{1}H NMR spectrum of ligand L measured in DMSO-d_6. 5

Figure S3. ^{13}C NMR spectrum of ligand L measured in DMSO-d_6. 6

Figure S4. ATR-FT-IR of ligand L (black) and Fe(III) complex (red) 6

Figure S5. HR-ESI-MS of ligand L. 7

Figure S6. HR-ESI-MS of iron(III) complex. 7

Figure S7. DPV curves of the lined L (A) and Fe(III) complex (B). 8

Figure S8. Spectroelectrochemistry of the ligand L measured in an anhydrous 0.1 M solution of $TBAPF_6$ in dichloromethane as the supporting electrolyte versus Fc/Fc^+ with a photograph of the corresponding redox state color. 8

Experimental section

Material and methods:

All reagents and solvents were obtained from commercial suppliers (Aldrich and Fluorochem) and applied directly without further purification. Nuclear magnetic resonance (NMR) spectra were recorded on a Bruker Avance NEO 600 MHz instrument (Bruker BioSpin GmbH, Rheinstetten, Germany). Chemical shifts (δ) are expressed in ppm and referenced to the residual proton peaks of the corresponding deuterated solvents (DMSO-d_6: 2.50 ppm). High-resolution mass spectra were collected in positive ionization mode using a QTOF Impact HD mass spectrometer (Impact HD Bruker, Bruker Daltonics, Bremen, Germany). UV-Vis-NIR absorption measurements were carried out with a Jasco V-770 spectrophotometer (JASCO Corporation, Tokyo, Japan). FT-IR spectra were acquired on a Bruker FT-IR INVENIO R spectrophotometer (Ettlingen, Germany).

Electrochemical experiments were performed using a multichannel BioLogic VSP potentiostat (BioLogic Science Instruments SAS, Seyssinet-Pariset, France) in anhydrous dichloromethane containing 0.1 M tetrabutylammonium hexafluorophosphate ($TBAPF_6$) as the supporting electrolyte. Prior to use, dichloromethane was dried over activated 4 Å molecular sieves and thoroughly degassed by purging with argon for 20 min. Electrochemical measurements were carried out under an inert argon atmosphere. A platinum disk electrode functioned as the working electrode, a platinum wire served as the counter electrode, and a non-aqueous Ag/Ag^+ electrode was employed as the reference. Spectroelectrochemical measurements were conducted with the same potentiostat interfaced with a Jasco V-770 UV-Vis-NIR spectrometer (JASCO Corporation, Tokyo, Japan). For spectroelectrochemical measurements, a commercially available honeycomb platinum working electrode (Pine Research Instrumentation, Inc., Durham, NC, USA) was employed together with a non-aqueous Ag/Ag^+ reference electrode. The honeycomb electrode is a platinum mesh working electrode arranged in a honeycomb geometry on a support, which leaves a large fraction of the optical path unobstructed and enables simultaneous electrochemical control and in situ UV-Vis-NIR spectral acquisition through the cell. In this configuration, the incident light passes through the open areas of the mesh rather than through a bulk metal surface. The platinum electrode itself remained visually unchanged throughout the applied potential range and did not contribute to the observed color changes. Consequently, the spectral and visual changes reported in this work originate exclusively from redox transformations of the dissolved analyte. Ag/Ag^+ reference

electrode was prepared by inserting a polished silver wire into a glass capillary equipped with a porous frit and filled with an acetonitrile solution containing 0.01 M $AgNO_3$ and 0.1 M TBAP.

X-ray crystallography

Diffraction data were collected by the ω- and ϕ-scan technique, at 100(1) K, using graphite-monochromated CuK_α radiation (λ=1.54178 Å), on Bruker D8 Quest four-circle diffractometer (Bruker AXS GmbH, Karlsruhe, Germany). The data were corrected for Lorentz-polarization as well as for absorption effects.[1] Precise unit-cell parameters were determined by a least-squares fit of the reflections of the highest intensity, chosen from the whole experiment. The structures were solved with SHELXT[2] and refined with the full-matrix least-squares procedure on F^2 by SHELXL.[3] All non-hydrogen atoms were refined anisotropically. Hydrogen atoms were placed in idealized positions and refined as 'riding model' with isotropic displacement parameters set at 1.2 (1.5 for methyl groups) times U_{eq} of appropriate carrier atoms. The structure contained the residual electron density in the voids, three solvent – acetonitrile molecules have been successfully modelled, but for the other the attempts were unsuccessful, sothe SQUEEZE procedure was applied, and the results suggest presence of another highly disordered acetonitrile molecule in the asymmetric part of the unit cell.

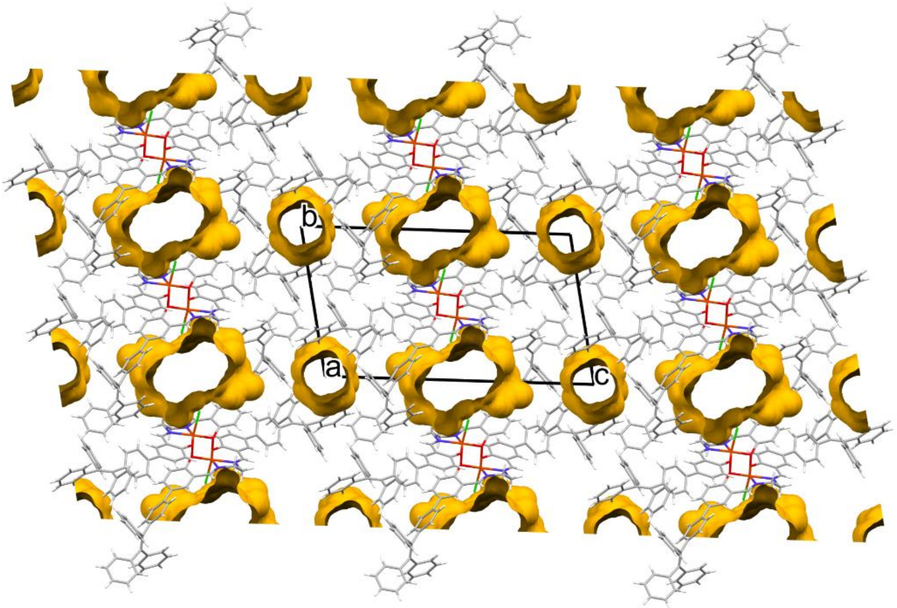

Figure S1. Voids in the structure 1 (after removing toluene molecule) as seen along b-direction.

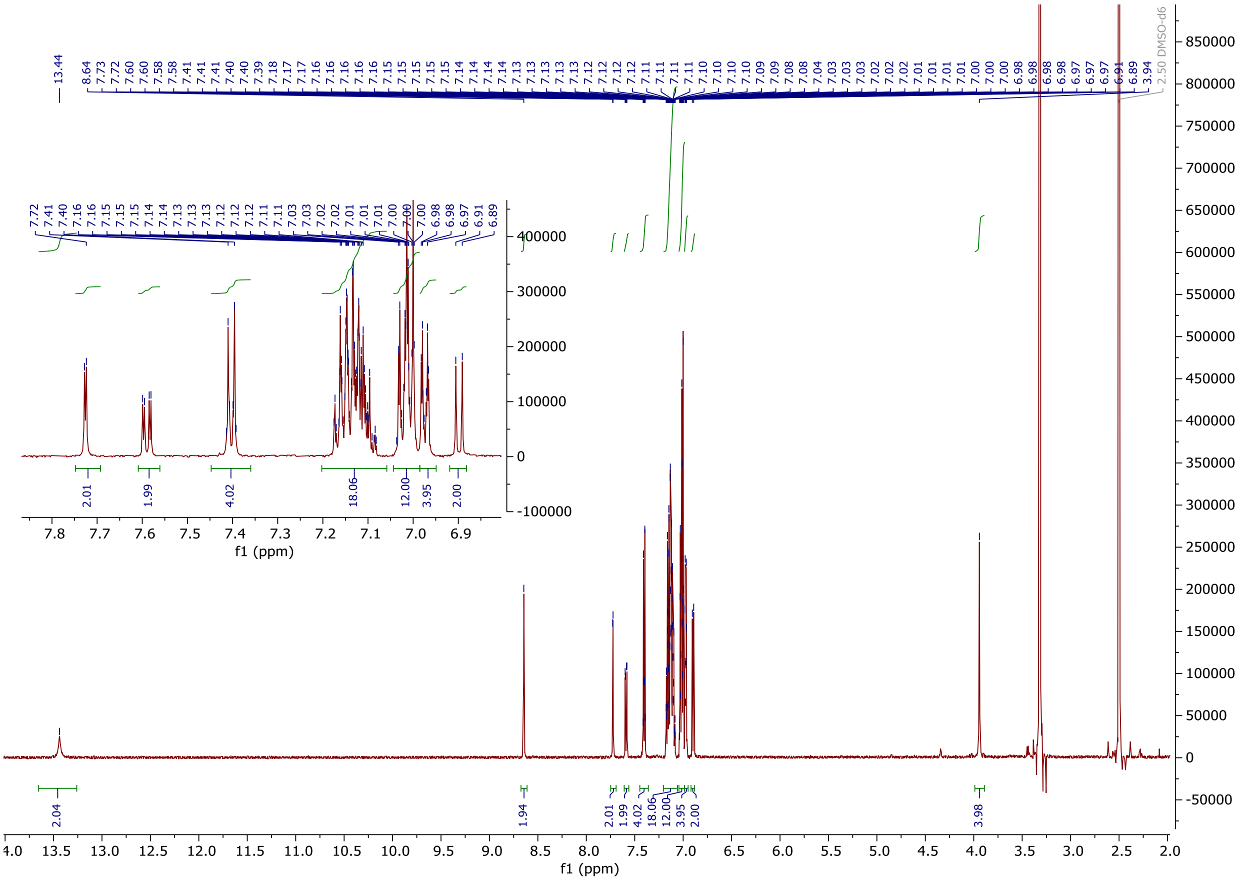

Figure S2. ^{1}H NMR spectrum of ligand **L** measured in DMSO-d$_6$.

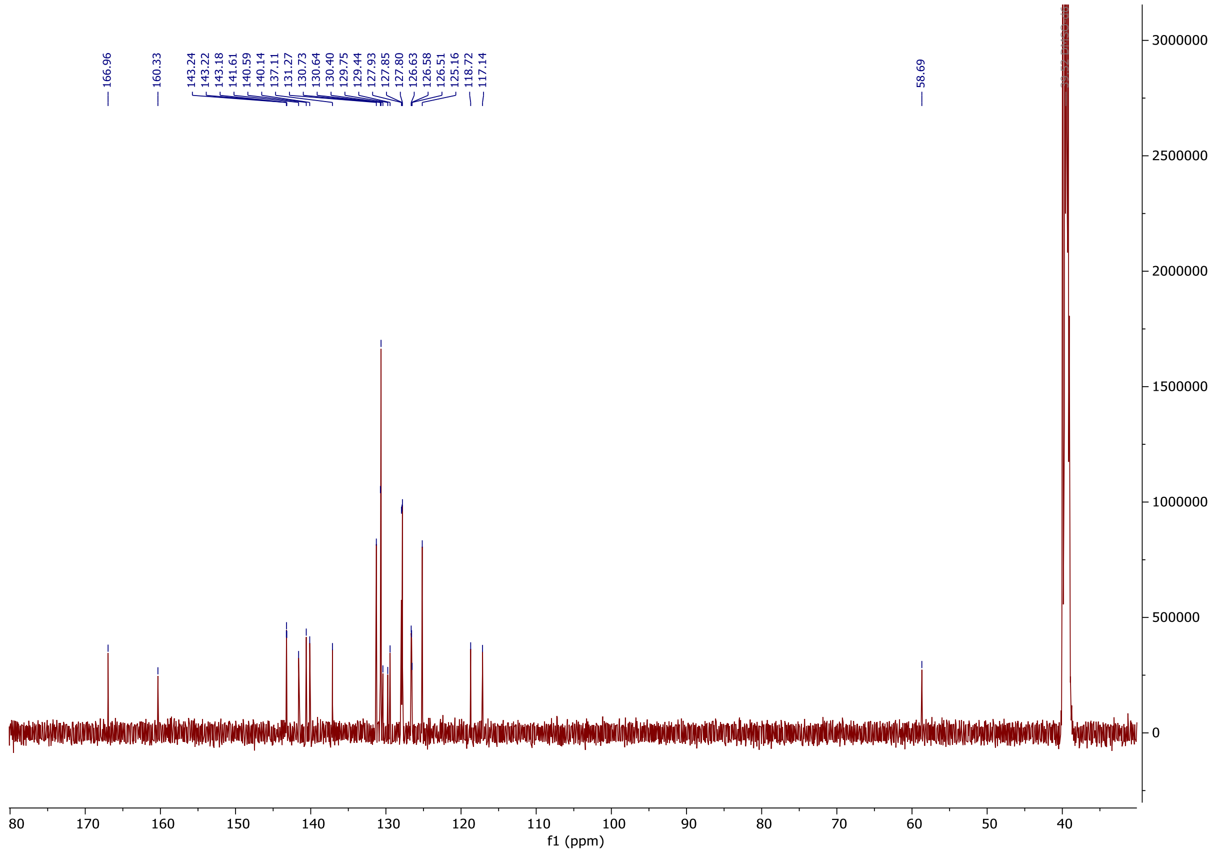

Figure S3. ^{13}C NMR spectrum of ligand **L** measured in DMSO-d$_6$.

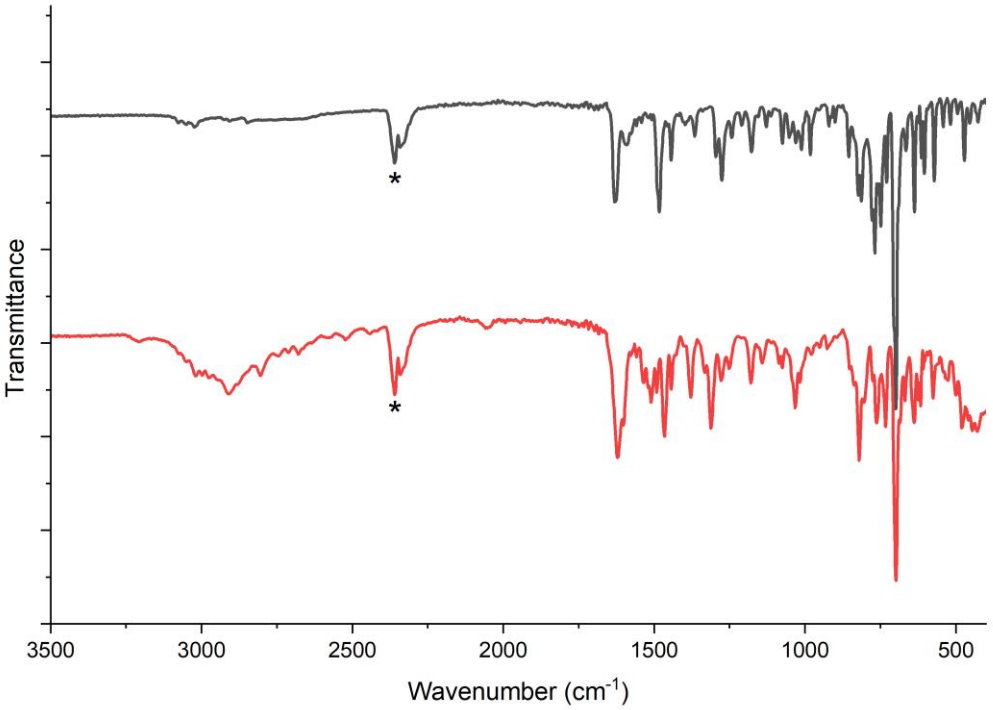

Figure S4. ATR-FT-IR of ligand L (black) and Fe(III) complex (red) (*indicates baseline correction artefacts).

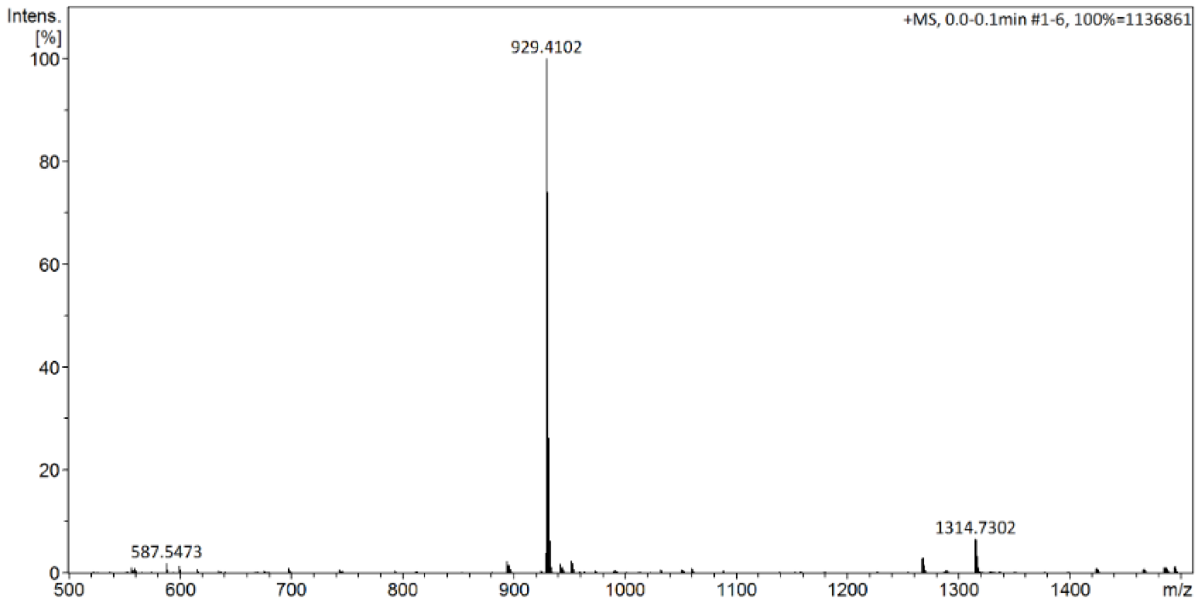

Figure S5. HR-ESI-MS of ligand **L**.

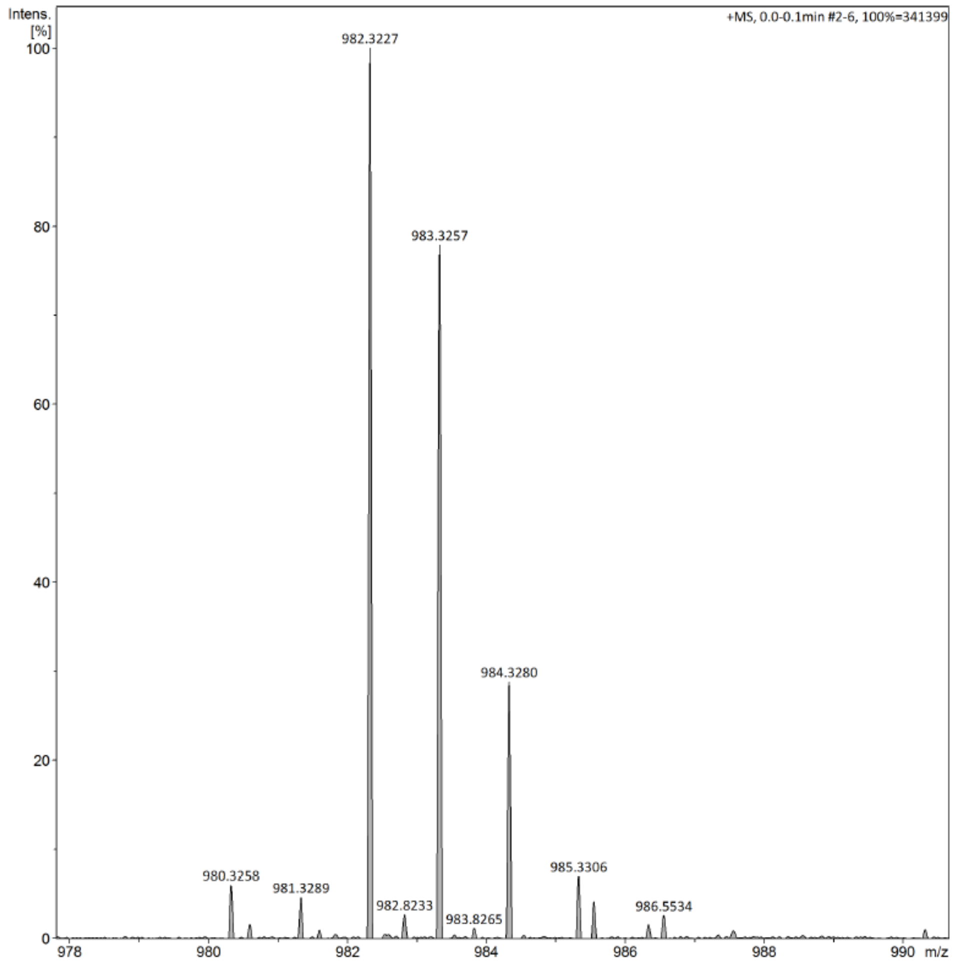

Figure S6. HR-ESI-MS of iron(III) complex.

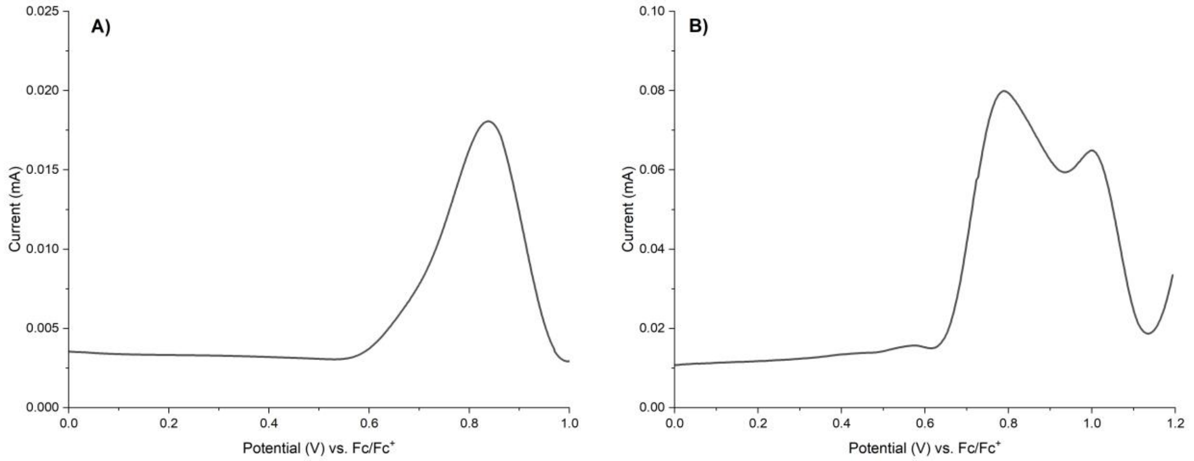

Figure S7. DPV curves of the lined L (A) and Fe(III) complex (B).

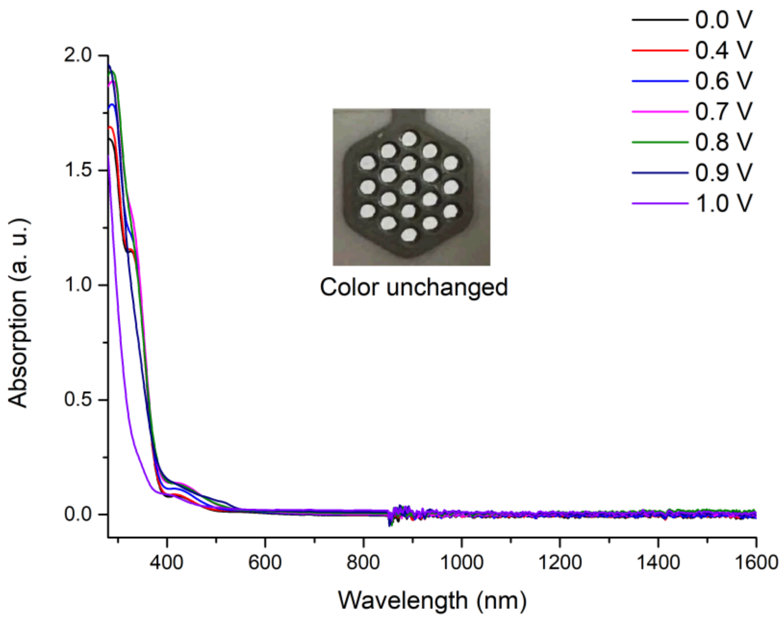

Figure S8. Spectroelectrochemistry of the ligand **L** measured in an anhydrous 0.1 M solution of $TBAPF_6$ in dichloromethane as the supporting electrolyte versus Fc/Fc^+ with a photograph of the corresponding redox state color.

References

[1] Rigaku Oxford Diffr. CrysAlis PRO (Version 1.171.41.113a).

[2] G.M. Sheldrick, *SHELXT* – Integrated space-group and crystal-structure determination, Acta Crystallogr. A Found. Adv. 71 (2015) 3–8. https://doi.org/10.1107/S2053273314026370.

[3] G.M. Sheldrick, Crystal structure refinement with *SHELXL*, Acta Crystallogr. C Struct. Chem. 71 (2015) 3–8. https://doi.org/10.1107/S2053229614024218.
